# Brainstem dysfunction induced by laser-induced shock wave results in hippocampal CA3 neuronal injury in mice

**DOI:** 10.3389/fneur.2025.1722482

**Published:** 2026-01-12

**Authors:** Tatsunori Nagamura, Soichiro Seno, Nobuaki Kiriu, Michiko Motegi, Satoko Kawauchi, Satoshi Tomura, Tetsuro Kiyozumi

**Affiliations:** 1 Department of Traumatology and Critical Care Medicine, National Defense Medical College, Saitama, Japan; 2 Division of Traumatology, Research Institute, National Defense Medical College, Saitama, Japan; 3 Division of Bioinformation and Therapeutic Systems, National Defense Medical College, Saitama, Japan

**Keywords:** brainstem, CA3 region, hippocampus, hypoxic brain injury, respiratory arrest, traumatic brain injury

## Abstract

The brainstem is anatomically vulnerable to trauma, and dysfunction often results in respiratory arrest and subsequent hypoxemia. Although animal models of traumatic brainstem injury have provided insights into local pathology, little is known about secondary effects, such as hypoxic brain injury, in other brain regions. Although a wide variety of traumatic brain injury models have been developed, few have been designed to replicate systemic hypoxemia as a primary insult. In this study, we reproduced brainstem dysfunction using laser-induced shock wave (LISW) and investigated their effect on hippocampal CA1 and CA3 pyramidal neurons, both of which are highly vulnerable to hypoxic injury. We applied LISWs to the upper neck region of mice, which caused immediate and transient respiratory arrest. Consequently, oxygen saturation rapidly declined and severe hypoxemia persisted for several minutes, reflecting transient brainstem dysfunction. Glial fibrillary acidic protein (GFAP) immunostaining revealed a significant increase in reactive astrocytes in both CA1 and CA3 regions on day 3. Iba-1 (ionized calcium-binding adapter molecule 1) immunostaining revealed no significant difference in the number of amoeboid microglia between the CA1 and CA3 regions. Cresyl violet staining revealed a time-dependent increase in the number of necrotic pyramidal neurons in the CA3 region, particularly on days 7 and 28. Terminal deoxynucleotidyl transferase dUTP nick-end labeling (TUNEL) staining demonstrated a significant increase in apoptotic cells in the CA3 region on day 1, indicating early-phase activation of apoptosis-related pathways prior to the emergence of delayed neuronal degeneration. These findings indicate that LISW-induced hypoxemia secondary to brainstem dysfunction selectively damages the CA3 pyramidal neurons. This model may provide a useful platform for studying brainstem dysfunction–induced hypoxemia and assessing potential therapeutic strategies.

## 1 Introduction

The brainstem is located caudally between the diencephalon, spinal cord, and cerebellum. It plays a crucial role in regulating the autonomic nervous system and integrating essential physiological functions such as respiratory regulation, cardiovascular control, and sensory processing (1). Due to its anatomical orientation along the rostro-caudal axis, the brainstem is particularly vulnerable to mild traumatic brain injury (TBI) (1). Epidemiological studies report a wide incidence of brainstem injury, ranging from 8.8 to 52% (2–4). Injury to the rostro-ventral medulla oblongata is associated with central respiratory arrest or ataxic breathing, which are markers of poor prognosis (5). In TBI, respiratory center dysfunction can occur either as a direct consequence of brainstem injury or due to increased intracranial pressure and mass effects associated with cerebral hemorrhage or edema (6).

To investigate these mechanisms, experimental models that reproduce brainstem dysfunction, particularly those involving respiratory centers, are needed. Although numerous TBI models have been developed, only a few have specifically addressed systemic hypoxemia as the primary insult. We developed a model by applying laser-induced shock wave (LISW) to the upper neck region of mice (7). LISW has several advantages, including compact size, ease of control, and high safety, making it suitable for targeted application in small animal models (8–10). Exposure of the upper neck region to LISW transiently induces brainstem dysfunction, resulting in immediate and reversible respiratory arrest. Shortly after exposure, oxygen saturation (SpO₂) decreases precipitously, leading to a hypoxemic state that lasts several minutes (7). Histopathological analysis reveals axonal injury in the medulla oblongata, as demonstrated by Bodian staining (7).

Because brainstem dysfunction can lead to respiratory arrest and hypoxemia, it is essential to evaluate secondary hypoxic brain injuries in remote regions. Previous animal models of brainstem injury have primarily focused on demonstrating pathological changes within the brainstem, whereas their impact on other brain regions has rarely been investigated. Because brain regions differ in their susceptibility to oxygen deprivation, some areas are particularly vulnerable to hypoxia. The hippocampus, especially the pyramidal neurons in the CA1 and CA3 regions, are known to be highly sensitive to hypoxic injury (11, 12). Hypoxia activates astrocytes and microglia, promotes the release of pro-inflammatory mediators, and contributes to neurodegeneration (13). In this study, we aimed to determine whether brainstem dysfunction–induced hypoxemia following LISW exposure leads to histopathological changes in hippocampal CA1 and CA3 neurons.

## 2 Methods

All animal experiments were conducted in accordance with the guidelines of the Institutional Review Board for the Care of Animal Subjects at the National Defense Medical College, Japan (approval number: 23054).

### 2.1 Animals

Eight-week-old male C57BL/6 mice, weighing 22–25 g, were obtained from SLC Japan (Shizuoka, Japan). The mice were housed at 22–24 °C with a 12-h light/dark cycle and had free access to food and water. Suffering was minimized by anaesthetizing the mice and employing humane endpoints. All experiments were performed in accordance with the relevant guidelines and regulations.

### 2.2 LISW

**Figure 1:**
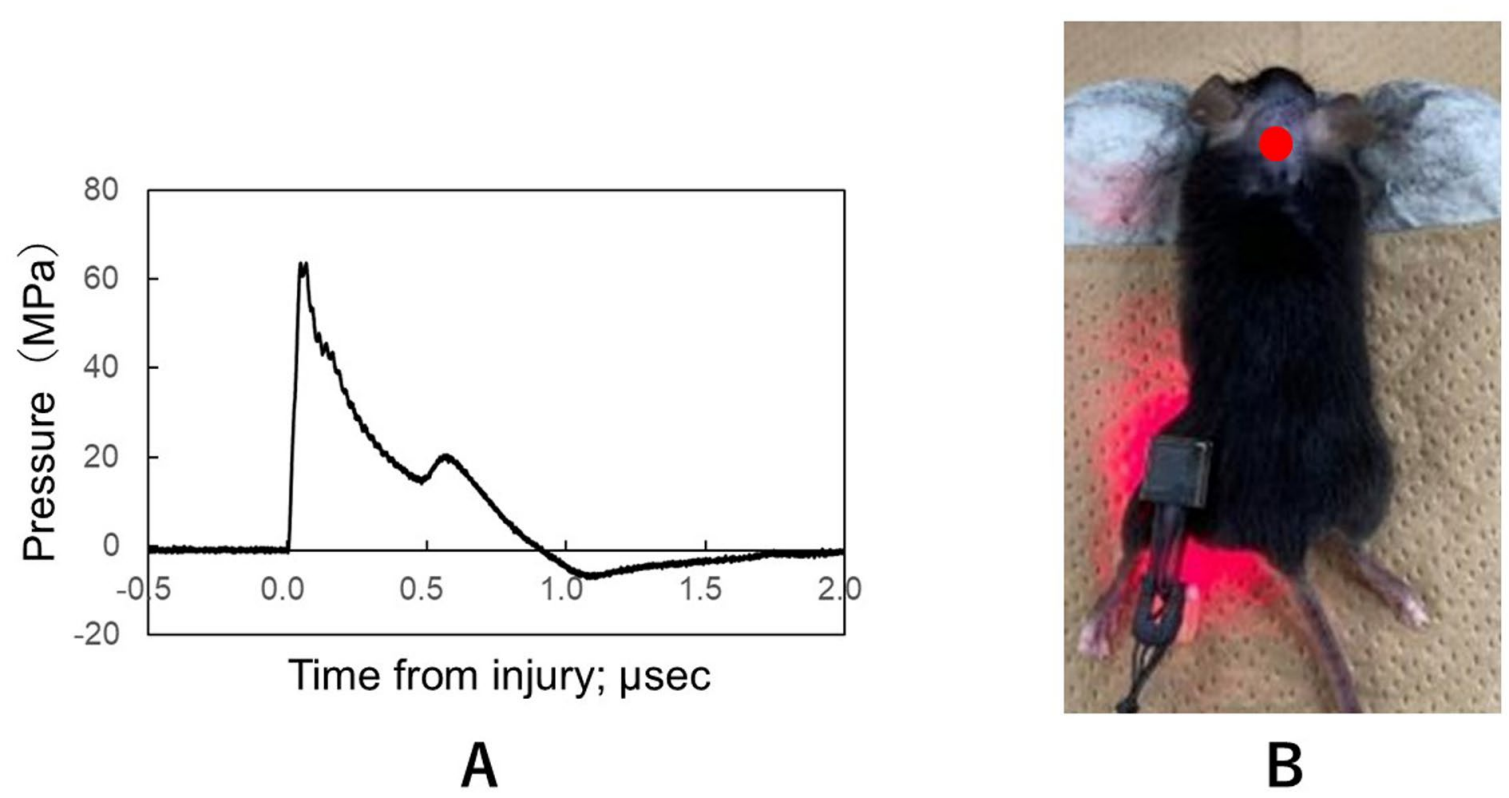
**(A)** Typical time–pressure waveform for LISWs with a laser fluence of 3.0 J/cm^2^: Peak pressure, 63.6 MPa; impulse, 19.8 Pa·s; duration, 0.89 μs. LISW, laser-induced shock wave. **(B)** Schema for the region of LISW exposure. A single pulse by LISW was applied on the skin surface of the upper neck region, 5 mm caudal to the line connecting the two ears. The laser target was 5-mm in diameter. LISW, laser-induced shock wave.

A Q-switched ruby laser (Ruby nano Q, NIIC Co., Tokyo, Japan; 694 nm; pulse width, 20 ns) was used as described previously (7). Briefly, the fluence of the LISW was 3.0 J/cm^2^, and the impulse of the LISW was 19.8 Pa·s (peak pressure, 63.6 MPa; Figure 1A). The experimental mice were anesthetized with an intramuscular injection of ketamine and xylazine. Hair on the upper dorsal neck was removed. The mice were placed on a plate in the supine position. A light-absorbing laser target (0.5-mm thick natural black rubber disk) bonded to an optically transparent material (1.0-mm thick polyethylene terephthalate sheet) was placed on the surface of the skin of the upper dorsal neck (Figure 1B). Subsequently, the ruby laser was used to deliver intense laser pulses to the laser target with the required amount of energy. The laser pulse was absorbed by the light-absorbing material and induced plasma, with its expansion generating shock waves. An intramuscular injection of buprenorphine hydrochloride was administered for pain relief after measuring the mouse’s vital signs. Sham-operated mice underwent the same procedures, including anesthesia and hair removal on the upper dorsal neck, but did not receive LISW exposure.

### 2.3 Physiological measurements

SpO₂ and respiratory rate (Mouse OX; STARR Life Science, Oakmont, PA, USA) were noninvasively monitored before and after LISW exposure in both LISW-exposed (*n* = 10) and sham mice (*n* = 5).

### 2.4 Tissue preparation

Histopathological analyses of mouse brains were performed at 0 (2 h), 3, 7, 14, 28 days after LISW exposure, and sham mice. The mice were deeply anesthetized with intramuscular injection of 100 mg/kg ketamine and 10 mg/kg xylazine, and transcardially perfused with normal saline followed by 4% paraformaldehyde. The brains were then excised and further fixed for 24 h in 4% paraformaldehyde before embedding in paraffin. Sagittal sections 1 mm lateral to the midline (5 μm thick) were prepared for hematoxylin and eosin (HE) staining, immunohistochemistry, Cresyl Violet staining, and terminal deoxynucleotidyl transferase dUTP nick-end labeling (TUNEL) staining to evaluate the left hippocampus. We selected sagittal rather than coronal sections because this orientation also enabled the direct assessment of shockwave propagation from the dorsal cerebellum, located immediately beneath the LISW exposure site. The hippocampal morphology at this level closely resembled that observed in coronal sections, allowing clear identification of the CA1, CA3, and dentate gyrus (DG) subfields (Supplementary Figure 1). Based on these considerations, and in line with previous studies using sagittal sections for hippocampal evaluation (14, 15), we believe that this approach provides a valid and reliable assessment of hippocampal injury in our model.

### 2.5 Immunohistochemical staining

For immunohistochemical staining, the sections were de-paraffinized and hydrated. For antigen retrieval, these sections were autoclaved using a Decliaking ChamberTM (Biocare Medical) with 10 mM citrate buffer (pH 6.0, Emergo Europe) for 10 min at 110 °C. Endogenous peroxidases were blocked using 3% hydrogen peroxide in methanol for 5 min. Further incubation with 10% goat serum (Nichirei Biosciences, Tokyo, Japan) was performed for 30 min at 37 °C. Sections were then incubated overnight at 4 °C with primary antibodies against glial fibrillary acidic protein (GFAP; 1:500, Abcam, Cat# ab7260, RRID:AB_305808) and ionized calcium-binding adapter molecule 1 (Iba-1; 1:20,000, Abcam, Cat# ab178846, RRID:AB_2636859). After washing with phosphate-buffered saline, sections were incubated with N-Histofine® Simple Stain Mouse MAX-PO (Nichirei Biosciences, Tokyo, Japan) for 30 min at room temperature. The immunoreactivity was visualized using diaminobenzidine (Histofine; ®, Nichirei Biosciences). The numbers of reactive astrocytes (GFAP-positive) and amoeboid microglia (Iba-1-positive) in the CA1 and CA3 regions of the left hippocampus were counted under 40 × magnification using a BZ-X710® microscope (Keyence Corporation, Osaka, Japan). The mean values from three representative sections (270 × 270 μm each) were used for analysis.

### 2.6 Cresyl violet staining

Sections for cresyl violet staining were de-paraffinized, rehydrated, and incubated in 0.1% cresyl violet solution (ab246816, Abcam) for 15 min at room temperature. After rinsing with distilled water for 1 min, the sections were differentiated in 95% alcohol with 10% acetic acid for 20 s, then 95% alcohol for 1 min. The sections were dehydrated with 100% alcohol and xylene.

The number of pyramidal necrotic neurons in the left CA1 and CA3 region were counted using BZ-X710® under 40 × magnifcation, and the average of three sections was used for the analysis. Pyramidal neurons that appeared triangular or irregular in outline and whose nuclei appeared pyknotic, were counted as necrotic neurons (16).

Based on a previous study reporting a large difference in necrotic neuron counts between the sham and day 3 groups (approximately 3 vs. 30 neurons, respectively; standard deviation [SD] = 3) (16), we estimated that a sample size of 6–8 animals per group would provide sufficient power (*α* = 0.05, power = 0.8). This sample size estimation was also applied to the GFAP and Iba-1 analyses because these measurements were performed using the same specimens and were expected to yield comparably detectable differences. Anticipating potential dropouts and aiming to strengthen statistical robustness, we ultimately used 10 animals per group.

### 2.7 TUNEL staining

TUNEL assay was performed as an additional experiment to assess apoptosis in the hippocampal CA1 and CA3 regions. The assay was conducted on the same hippocampal sections using a fluorescein cell death detection kit (NBP3-11957, RSD). TUNEL-positive signals were quantified within standardized regions of interest (350 × 350 μm) in the left CA1 and CA3 areas using a fluorescence microscope (BZ-X710®) under 20 × magnification. Four groups (sham, day 1, day 3, and day 7) were analyzed, with five animals per group, which is within the typical range used for histological apoptosis assays. Based on previous reports that TUNEL signals peak within 24–48 h after experimental traumatic brain injury (17, 18), we included a day 1 time point to better capture the acute phase of cell death. We additionally compared CA1 and CA3 regions on day 1 to evaluate region-specific vulnerability during the early post-injury period.

### 2.8 Behavioral assessment

We examined short-term memory in the sham and day 28 groups (*n* = 10 per group) using the Y-Maze Spontaneous Alternation Test as previously described (10). Briefly, the symmetrical Y-maze consists of three acrylic arms (25 × 5 cm) positioned 120° apart with 30-cm-high transparent walls. Each mouse was placed at the center and allowed to freely explore for 8 min. The sequence and total number of arm entries were recorded, and the percentage of spontaneous alternation was calculated as follows: (Number of Alternations / [Total number of arm entries - 2]) × 100 (19).

### 2.9 Statistical analysis

Data are expressed as means ± SDs. The numbers of reactive astrocytes, amoeboid microglia, necrotic neurons, and TUNEL positive areas across the four groups (sham vs. day 1 vs. day 3 vs. day 7) were analyzed using the one-way factorial analysis of variance with post-hoc comparisons and Tukey’s test. Vital signs and TUNEL positive areas between CA1 and CA3 on day 1 were analyzed using unpaired t-tests. Statistical significance was set at a *p*-value of less than 0.05. Statistical analyses were performed using SPSS version 24.0 for Windows (IBM Corp., Armonk, NY, USA).

## 3 Results

### 3.1 Physiological findings

A total of 62 mice were subjected to LISW exposure, of which 12 died within several minutes after exposure (mortality rate: 19.4%). The remaining 50 mice were included in the final analysis (10 mice each at day 0, 3, 7, 14, and 28). In addition, five mice were used for the day 1 TUNEL analysis as part of an additional apoptosis assessment.

#### 3.1.1 Vital signs

**Figure 2:**
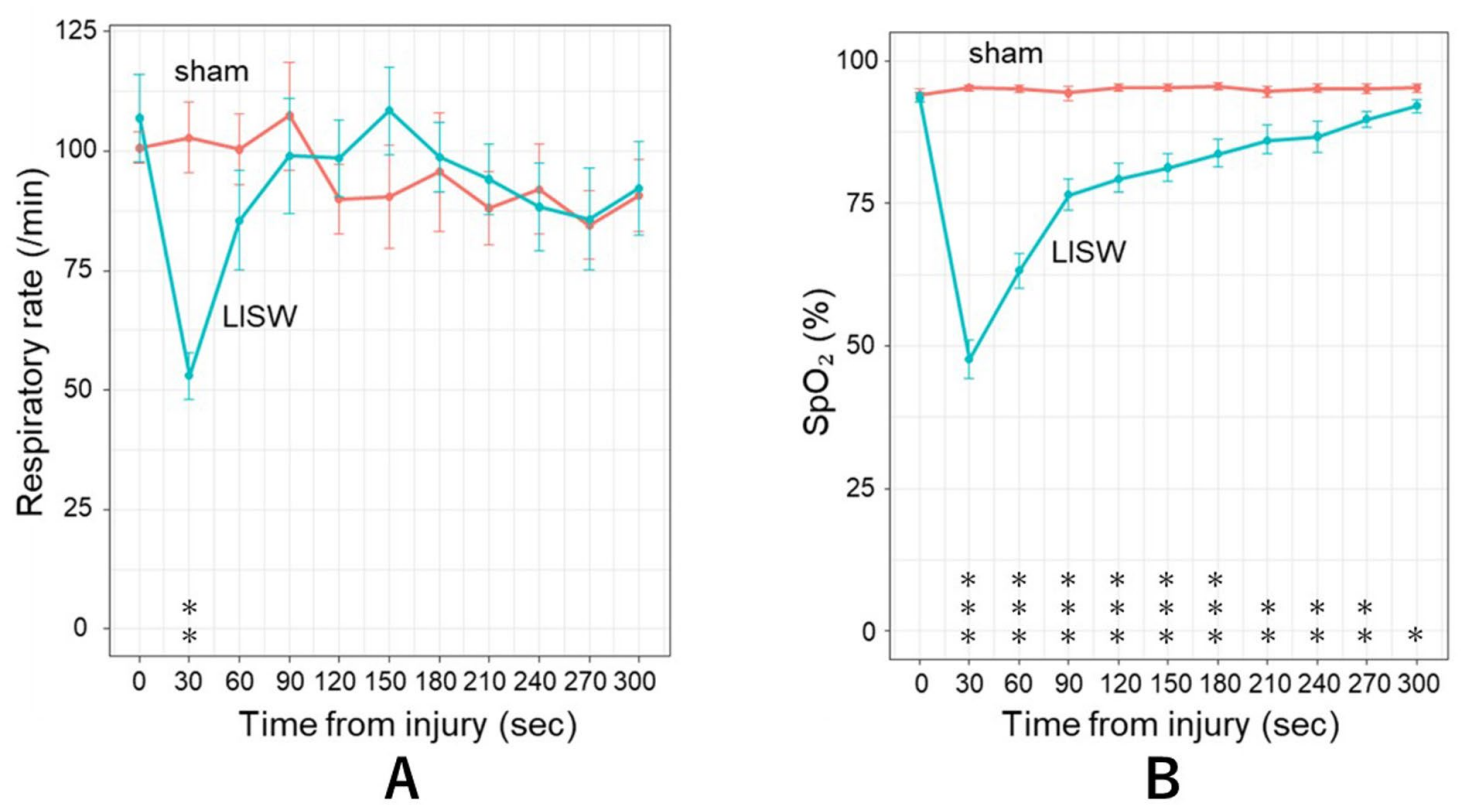
**(A)** Respiratory rate before and after LISW exposure. LISW exposure to the upper neck region caused immediate respiratory arrest in mice. Breathing resumed within 10 to 20 s and gradually returned to baseline by approximately 120 s post-injury. The respiratory rate at 30 s after LISW was significantly decreased compared with that in sham mice (52.8 ± 15.1 vs. 106.8 ± 29.2 breaths/min, respectively, *p* < 0.01). LISW, laser-induced shock wave. **(B)** Oxygen saturation (SpO₂) before and after LISW exposure. SpO₂ dropped immediately after LISW exposure, rapidly decreasing to 47.7 ± 10.4% at 30 s compared with that in sham mice (95.2 ± 1.3%, *p* < 0.001). SpO₂ gradually recovered and returned to baseline levels by approximately 300 s post-injury. Hypoxemia persisted for about 300 s following the injury. LISW, laser-induced shock wave.

When the LISWs were applied to the upper dorsal neck region of the mice, breathing stopped immediately. Most mice demonstrate opisthotonic posturing, suggesting that the shock wave had reached the brainstem and induced transient brainstem dysfunction. Their breathing restarted within 10–20 s and returned to baseline approximately 120 s after the injury. The respiratory rate of LISW mice at 30 s was significantly lower than sham mice (LISW: 52.8 ± 15.1 breaths/min; sham: 106.8 ± 29.2 breaths/min; *p* < 0.01; Figure 2A). Moreover, the SpO₂ of LISW mice dropped immediately after injury, rapidly falling to 40% at 30 s (LISW: 47.7 ± 10.4%; sham: 95.2 ± 1.3%; *p* < 0.01; Figure 2B). It returned to baseline approximately 300 s after injury. Hypoxemia was observed for approximately 300 s during this period.

### 3.2 Histopathological findings

#### 3.2.1 HE staining

On day 0, all mice had subdural hematomas over the surface areas extending from the occipital lobe to the cerebellum. This hematoma was likely due to injury to the cortical veins near the impact site. Residual hematomas were detected in seven of 10 mice on day 14 and in four of 10 mice on day 28. Four of the 50 mice had brain contusions around the same surface area. There was no hemorrhage in other areas, such as the hippocampus or brainstem (Supplementary Figure 2).

#### 3.2.2 GFAP immunohistochemistry

**Figure 3:**
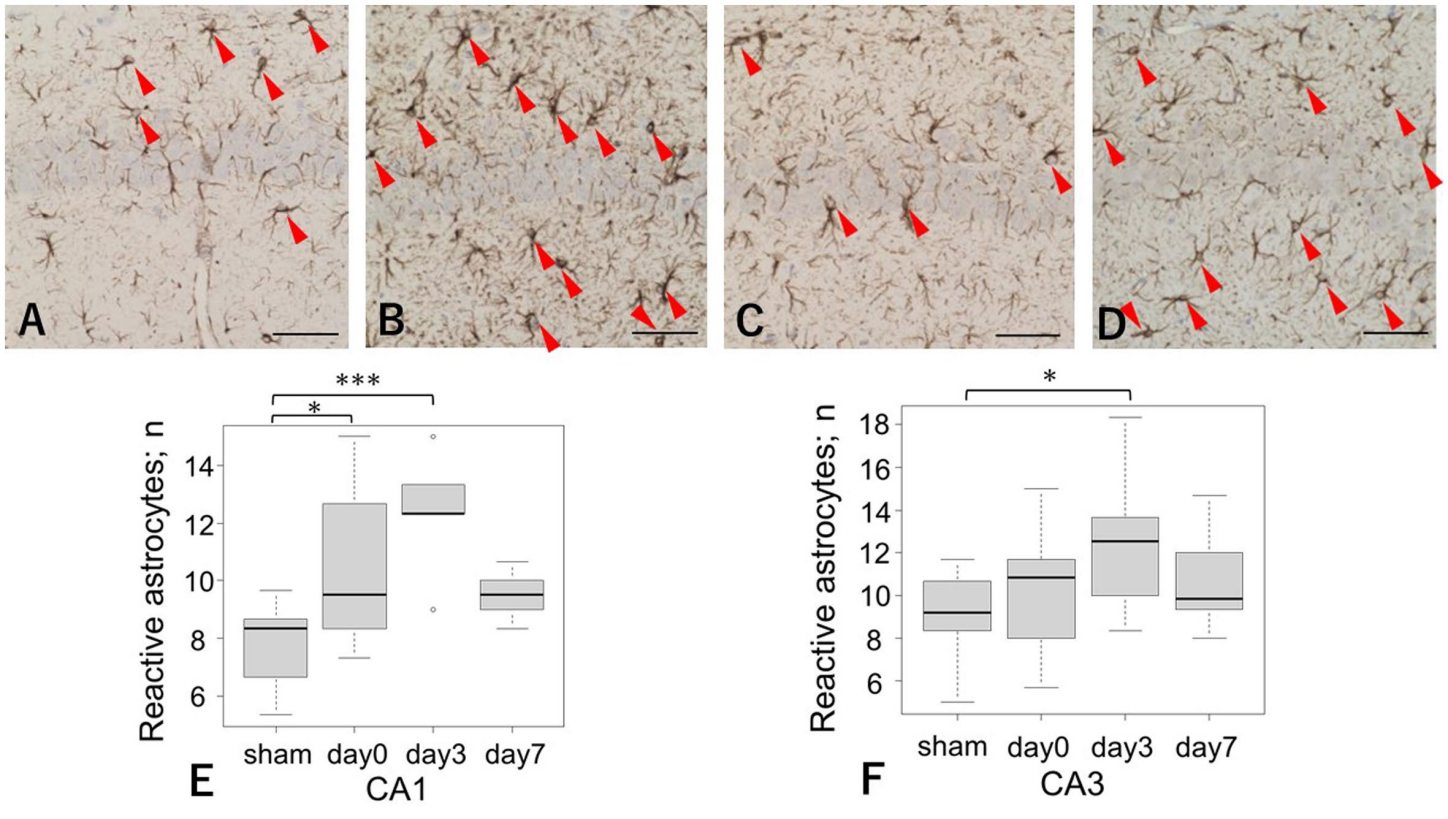
GFAP immunoreactivity in the CA1 and CA3 regions after LISW exposure. **(A–D)** Representative images of GFAP-positive astrocytes infiltrating the pyramidal cell layers. Arrows indicate reactive astrocytes. Scale bars = 50 μm. *: *p* < 0.05, ***: *p* < 0.001. GFAP, glial fibrillary acidic protein. **(A)** Sham in the CA1 region; **(B)** Day 3 post-injury in the CA1 region; **(C)** Sham in the CA3 region; **(D)** Day 3 post-injury in the CA3 region. **(E)** Quantification of reactive astrocytes in the CA1 region. The number of reactive astrocytes was significantly higher on day 3 than in the sham group. **(F)** Quantification of reactive astrocytes in the CA3 region. Day 3 showed a significantly greater number of reactive astrocytes compared with the sham group.

The number of reactive astrocytes on day 0 was significantly higher in the LISW group than in the sham group, specifically in the CA1 region (10.4 ± 2.7 vs. 7.9 ± 1.3, respectively, *p* = 0.04). The number of reactive astrocytes on day 3 was also significantly higher in the LISW group than in the sham group, with increases observed in both the CA1 and CA3 regions (CA1: 12.5 ± 1.5 vs. 7.9 ± 1.3, respectively; *p* < 0.01; CA3: 12.4 ± 2.1 vs. 9.1 ± 1.9, respectively; *p* = 0.04). In contrast, no apparent increase in the number of reactive astrocytes was observed in the brainstem (Figure 3).

#### 3.2.3 Iba-1 immunohistochemistry

**Figure 4:**
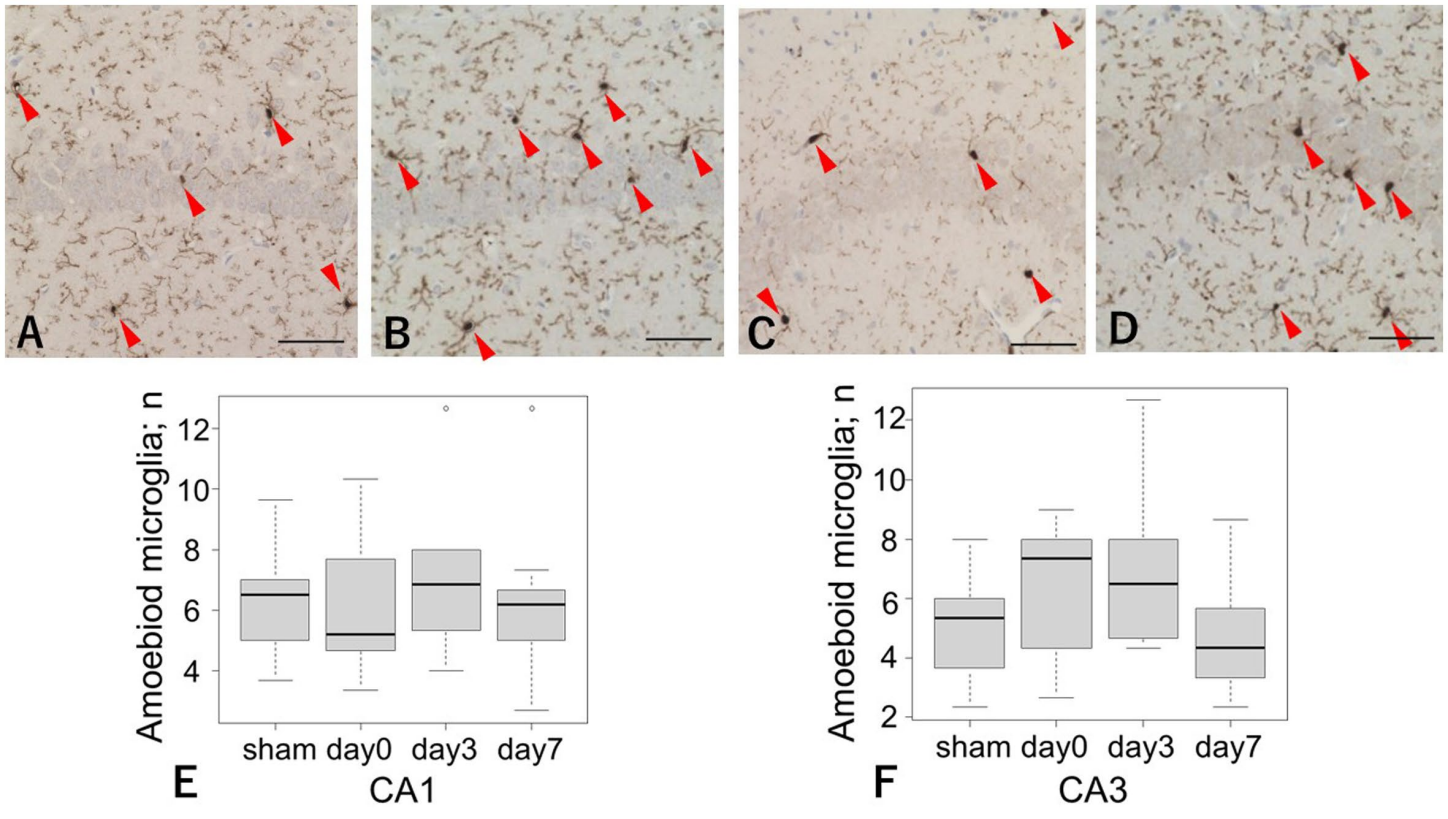
Iba-1 immunoreactivity in the CA1 and CA3 regions after LISW exposure. **(A–D)** Representative images of Iba-1-positive microglia infiltrating the pyramidal cell layers. Arrows indicate amoeboid microglia. Scale bars = 50 μm. Iba-1, ionized calcium-binding adapter molecule 1; LISW, laser-induced shock wave. **(A)** Sham in the CA1 region; **(B)** Day 3 post-injury in the CA1 region; **(C)** Sham in the CA3 region; **(D)** Day 3 post-injury in the CA3 region. **(E)** Quantification of amoeboid microglia in the CA1 region. There was no significant difference in the number of amoeboid microglia between the sham and day 3 groups. **(F)** Quantification of reactive astrocytes in the CA3 region. There was no significant difference in the number of amoeboid microglia between the sham and day 3 group.

There was no significant difference in the number of amoeboid microglia between the CA1 and CA3 regions (CA1: sham vs. day 3 = 6.3 ± 1.7 vs. 6.9 ± 2.5, respectively; *p* = 0.84; CA3: sham vs. day 3 = 5.0 ± 1.8 vs. 6.9 ± 1.9, respectively; *p* = 0.07). This result was not statistically significant; however, there was a slight upward trend in the CA3 region, which was mainly observed in the day 3 group. Similarly, no increase in the number of activated microglia was observed in the brainstem (Figure 4).

#### 3.2.4 Cresyl violet staining

**Figure 5:**
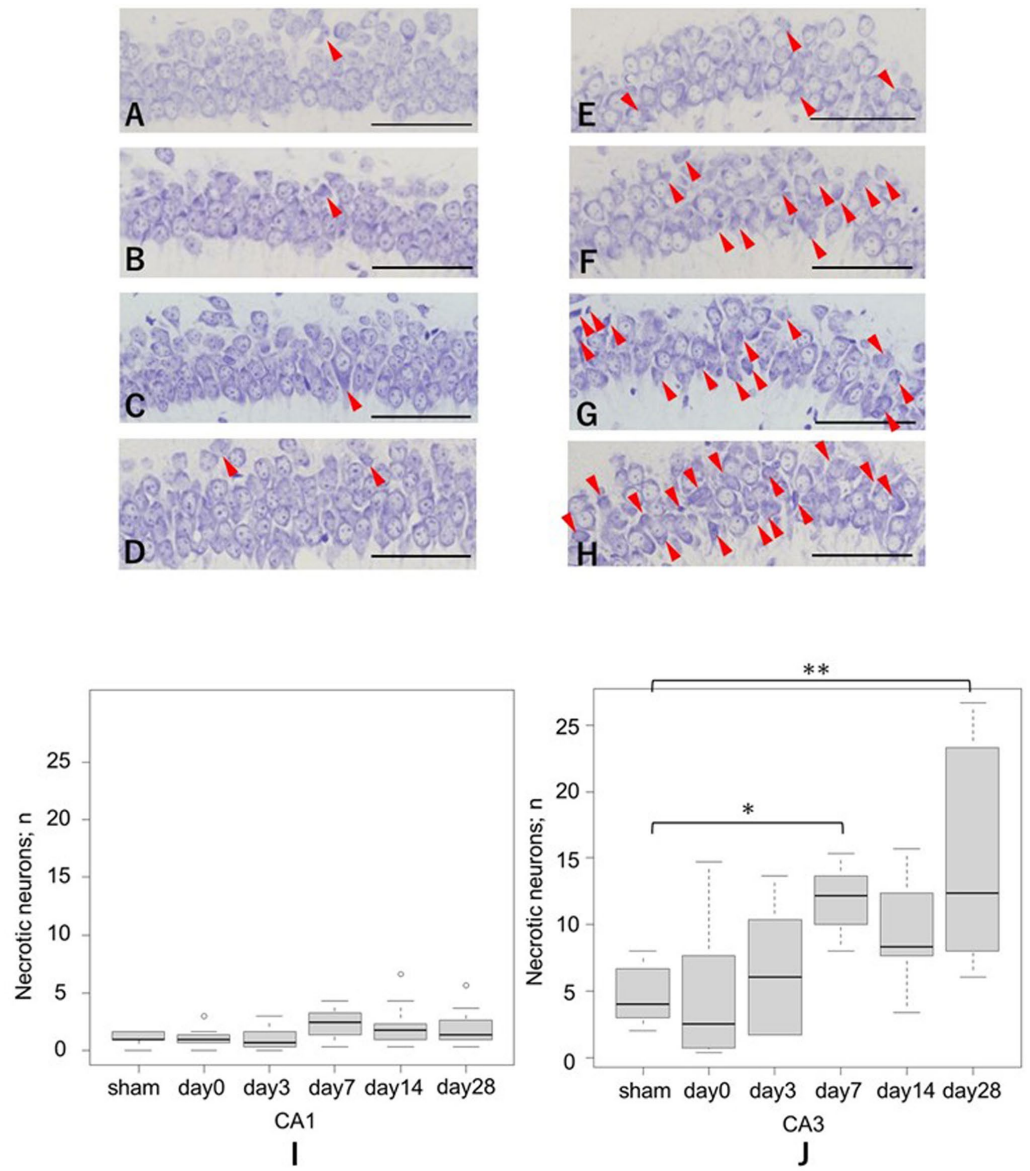
Quantification of necrotic neurons in the hippocampal CA1 and CA3 regions following LISW exposure, assessed by cresyl violet staining. **(A–D)** Representative images of necrotic cells infiltrating the pyramidal cell layers. Arrows indicate necrotic neurons. Scale bars = 50 μm. *: *p* < 0.05, **: *p* < 0.01. LISW, laser-induced shock wave. **(A)** Sham in the CA1 region; **(B)** Day 3 post-injury in the CA1 region; **(C)** Day7 post-injury in the CA1 region; **(D)** Day 28 post-injury in the CA1 region; **(E)** Sham in the CA3 region; **(F)** Day 3 post-injury in the CA3 region; **(G)** Day 7 post-injury in the CA3 region; **(H)** Day 28 post-injury in the CA3 region. **(I)** Quantification of necrotic cells in the CA1 region. No significant differences were observed in the CA1 region. **(J)** Quantification of necrotic cells in the CA3 region. The number of necrotic cells was significantly higher in the CA3 region on both days 7 and 28 compared with the sham group.

The number of necrotic neurons was significantly higher in the CA3 region of the LISW group than in the sham group on days 7 and 28, whereas no significant difference was observed in the CA1 region. In the CA3 region, the average number of necrotic neurons increased from 4.7 ± 2.2 in the sham group to 11.9 ± 2.4 on day 7 (*p* = 0.02), and to 15.0 ± 8.0 on day 28 (*p* < 0.01). In contrast, in the CA1 region, the changes from 1.1 ± 0.5 (sham) to 2.4 ± 1.5 on day 7 (*p* = 0.22) and to 1.9 ± 1.6 on day 28 (*p* = 0.68) were not statistically significant (Figure 5).

#### 3.2.5 TUNEL staining

**Figure 6:**
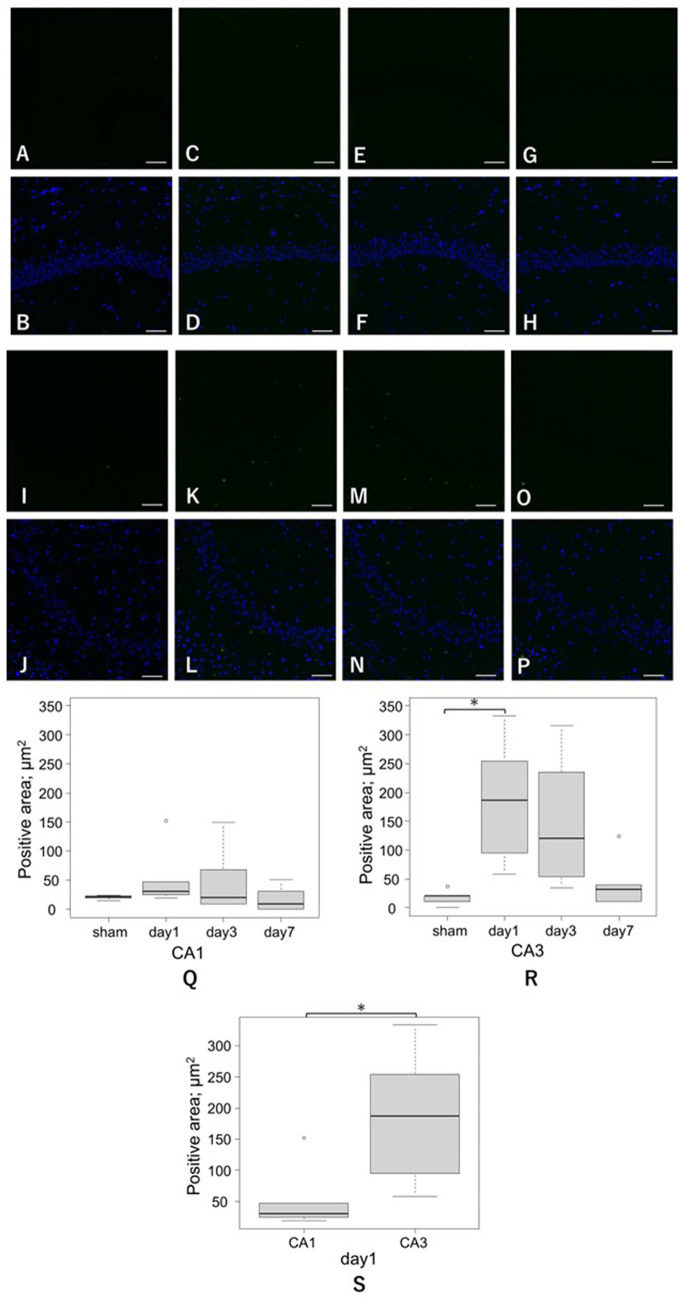
Representative TUNEL staining in the hippocampal CA1 and CA3 regions after LISW exposure. Scale bars = 50 μm. *: *p* < 0.05. TUNEL, terminal deoxynucleotidyl transferase dUTP nick-end labeling. **(A–H)** Representative images from the CA1 region: **(A,B)** sham, **(C,D)** day 1, **(E,F)** day 3, and **(G,H)** day 7; and **(A,C,E,G)** TUNEL staining, and **(B,D,F,H)** merged images of TUNEL and nuclear counterstaining. **(I–P)** Representative images from the CA3 region: **(I,J)** sham, **(K,L)** day 1, **(M,N)** day 3, and **(O,P)** day 7; and **(I,K,M,O)** TUNEL staining, and **(J,L,N,P)** merged images of TUNEL and nuclear counterstaining. **(Q)** Quantification of the TUNEL-positive area in the CA1 region across the four groups (sham, day 1, day 3, and day 7). No statistically significant differences were detected (sham vs. day 1 = 20.2 ± 4.0 μm^2^ vs. 54.8 ± 55.3 μm^2^, *p* = 0.58; sham vs. day 3 = 20.2 ± 4.0 μm^2^ vs. 51.0 ± 59.9 μm^2^, *p* = 0.67). **(R)** Quantification of the TUNEL-positive areas in the CA3 region. A significant increase was observed on day 1 compared with the sham group (sham vs. day 1 = 18.2 ± 14.0 μm^2^ vs. 185.4 ± 112.8 μm^2^, *p* = 0.03), indicating acute-phase enhancement of apoptosis-related signals. TUNEL-positive cells were present throughout the CA3 region, including both pyramidal neurons and surrounding non-pyramidal cells. **(S)** Quantification of the TUNEL-positive areas between CA1 and CA3 on day 1. At the acute peak, CA3 exhibited a significantly higher TUNEL-positive area than CA1 (185.4 ± 112.8 μm^2^ vs. 54.8 ± 55.3 μm^2^, respectively, *p* = 0.04).

The CA1 region showed no statistically significant differences in TUNEL-positive area among the four groups. In contrast, the CA3 region exhibited a significant increase on day 1 compared with the sham group (185.4 ± 112.8 μm^2^ vs. 18.2 ± 14.0 μm^2^, respectively, *p* = 0.03). Notably, TUNEL-positive cells were observed not only in pyramidal neurons but also in surrounding non-pyramidal cells, contributing to the overall increase in the TUNEL-positive area (Figure 6).

#### 3.2.6 Behavioral assessment

There was no statistically significant difference in the % Alternation between the sham and day 28 groups (59 ± 12% vs. 50 ± 10%, respectively, *p* = 0.08). Although this difference did not reach statistical significance, mice in the day 28 group exhibited a lower trend in the % Alternation, suggesting a potential decline in short-term spatial working memory (Supplementary Figure 3).

## 4 Discussion

We established a brainstem dysfunction model by applying LISW to the upper neck region of mice. This injury caused immediate respiratory arrest, followed by approximately 5 min of hypoxemia. This acute physiological insult led to distinct histopathological changes in the hippocampus: early apoptotic signals appeared in the CA3 region on day 1, reactive astrocytes peaked on day 3, and necrotic pyramidal neurons accumulated progressively thereafter. These findings suggest that hypoxemia secondary to brainstem dysfunction provokes apoptosis and astrogliosis, ultimately leading to the selective vulnerability of CA3 pyramidal neurons.

Two potential mechanisms could explain the selective vulnerability of the CA3 region observed in our model. First, CA3 neurons exhibit higher metabolic activity and oxygen consumption than CA1 neurons, which may make them more susceptible to hypoxia-induced metabolic stress (20). Second, CA3 neurons contain fewer calcium-binding proteins than CA1 neurons, thereby reducing the intracellular calcium buffering capacity and increasing susceptibility to excitotoxic and oxidative injury (21). Experimental models characterized by decreased SpO₂, such as post-traumatic hypoxia and hypobaric hypoxia, also support our observation that CA3 neurons are particularly vulnerable to LISW-induced hypoxemia (22–25).

The hippocampal CA3 damage observed in this model is likely attributable to hypoxemia rather than to direct mechanical damage from the shock wave. This interpretation was supported by histological comparisons between LISW applied to the upper neck and parietal regions (Supplementary Figure 4), which demonstrated distinct patterns of GFAP expression depending on the application site. When the LISW was applied to the brain, GFAP expression increased both at and beneath the impact site, with deeper infiltration observed at higher energy levels. In the parietal group, GFAP expression increased not only in the hippocampus, but also in the overlying parietal cortex (Supplementary Figures 4A–C). In contrast, LISW applied to the upper neck region increased GFAP expression, primarily in the dorsal cerebellum and caudal occipital cortex, with a minimal signal in the parietal region surrounding the hippocampus (Supplementary Figures 4D–F). Although previous *in vitro* studies have shown that astrocytes can undergo delayed morphological alterations and phagocytosis-like activity following LISW exposure (26), implying that some degree of direct mechanical influence cannot be completely excluded, such effects appear limited in our model. Moreover, non-brainstem (parietal) LISW exposure did not induce respiratory arrest or marked SpO₂ reduction (7), indicating that severe hypoxemia occurs only when brainstem dysfunction is induced. Therefore, the predominant pathological mechanism underlying hippocampal GFAP infiltration in this model is oxygen deprivation rather than direct tissue trauma from the shock wave.

Residual subdural hematomas persisting beyond the acute phase may also have contributed to hippocampal CA3 injury. The inflammatory response and iron deposition associated with these hematomas could have played a role in the astrogliosis observed in the hippocampal CA1 and CA3 regions at later time points. Notably, hemosiderin deposition was observed near the CA3 region in some mice (Supplementary Figure 5), suggesting that iron-mediated oxidative stress may act as an additional driver of astrogliosis. This interpretation is supported by previous studies demonstrating that iron overload can induce glial activation through oxidative stress–related pathways (27–29).

In this study, the number of reactive astrocytes significantly increased in the CA1 region on day 0. This finding may reflect the early astrocytic responses in the CA1 region. Astrocytes respond to decreases in the partial pressure of oxygen, a few millimeters of mercury below normal brain oxygenation, with elevations in intracellular calcium concentration (30). Under ischemic hypoxia, GFAP-positive astrocyte activation appears in the CA1 region as early as 3–6 h after the insult (31). While GFAP-positive astrocytes become particularly prominent in the CA1 region (32), a significant increase in their number in the CA3 region occurs only after severe ischemia (33). Moreover, GFAP-positive astrocytes have been reported to be more pronounced in the CA1 region than in the CA3 region under hypobaric hypoxia (34). Such region-specific astrocyte activation is consistent with the notion that astrocytes in the CA1 and CA3 subfields exhibit qualitatively distinct responses to ischemia and hypoxemia.

As a comparison with previous studies, a prior study using the COBIA (Cranium Only Blast Injury Apparatus) model in rats examined shock wave exposure to the brainstem region via the foramen magnum (35). They reported immediate respiratory arrest with sustained hypoxemia and microglial activation in the hippocampus, whereas our model showed significant astrocytic activation. This result may reflect differences in the evaluation methods (immunofluorescence vs. immunohistochemistry) and regions of interest (DG vs. CA1/CA3). Similarly, a rabbit model of mechanical brainstem injury was established using a high-speed air gun and hammer device (36). This model reproduced respiratory arrest and local pathology; however, it did not address the hypoxemic effects in the hippocampus. These studies demonstrated high reproducibility; however, they also required a large and complex apparatus and raised safety and management concerns owing to the use of a gun device. Unlike these previous studies, we evaluated hippocampal injury at multiple time points, from the acute phase to 28 days after injury. Furthermore, cresyl violet staining allowed us to assess pyramidal cell damage specifically, which is a novel aspect of the present study.

Our model represents a novel form of hypoxemia caused by transient respiratory arrest following LISW exposure to the upper neck region resulting from brainstem dysfunction involving the respiratory center. This model is easy to establish and highly reproducible. Moreover, it may serve as a valuable tool for studying brainstem-mediated respiratory dysfunction. Furthermore, it provides a practical platform for investigating both the mechanisms and therapeutic interventions targeting hippocampal vulnerability, particularly in the CA3 region. Additionally, the Y-maze results demonstrated a trend toward impaired performance in the chronic phase, suggesting that this model may also be useful for exploring hippocampus-related behavioral alterations, including anxiety or depressive behavioral changes associated with hippocampal dysfunction.

This study had a few limitations. First, although hypoxemia was monitored using SpO₂, the absence of arterial blood gas analysis limited the detailed physiological characterization of hypoxic insult. Second, we did not evaluate the potential effects of hypoxemia on other vulnerable brain regions such as the thalamus and cerebral cortex. Third, only male mice were used in this study, which may limit the generalizability of the findings across sexes. Fourth, blood pressure was not monitored during the procedure, which may have affected SpO₂ levels. However, a previous study using the same LISW model reported no marked decrease in mean arterial pressure (7), suggesting that severe hypotension is unlikely to have contributed to the observed hypoxemia. Fifth, we did not include a “simple hypoxia control group.” Because LISW-induced hypoxemia is accompanied by transient brainstem dysfunction rather than uniform oxygen reduction, it is technically difficult to reproduce this complex condition with hypoxia alone. Sixth, although the distance between the LISW source and the skin surface was kept constant across all animals, we did not evaluate whether distance from the shock wave influenced cellular activation or neuronal death.

## 5 Conclusion

In this study, LISW exposure in the upper neck region induced transient respiratory arrest and hypoxemia, resulting in selective neuronal injury in the hippocampal CA3 region. These findings highlight the vulnerability of CA3 pyramidal neurons to hypoxemia secondary to brainstem dysfunction. This model may provide a practical and reproducible platform for investigating brainstem-mediated respiratory dysfunction and its impact on the hypoxia-sensitive regions of the brain.

## Data Availability

The raw data supporting the conclusions of this article will be made available by the authors, without undue reservation.
