# Supplementary figures and images for: Brainstem dysfunction induced by laser-induced shock wave results in hippocampal CA3 neuronal injury in mice
Source: Front Neurol. 2026 Jan 12;16:1722482. doi: 10.3389/fneur.2025.1722482 (PMC12832503; doi:10.3389/fneur.2025.1722482)

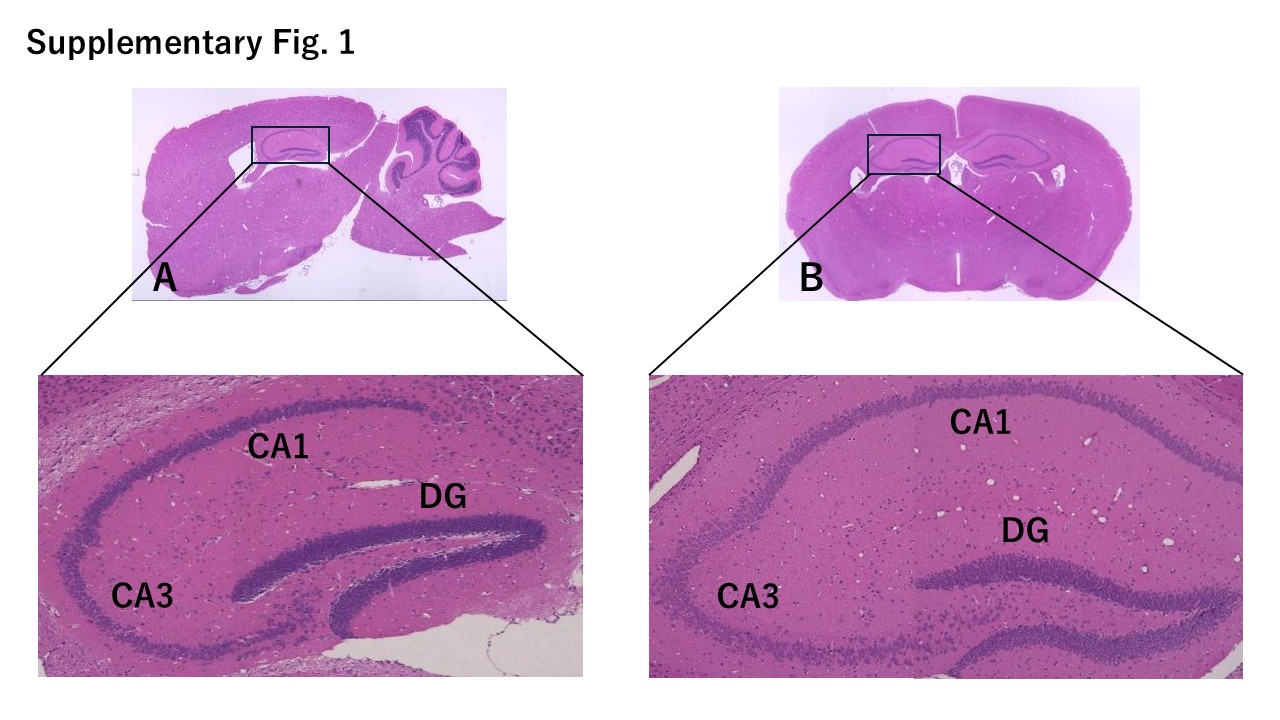

Supplement: Supplementary file 1 [file Image_1.jpeg]

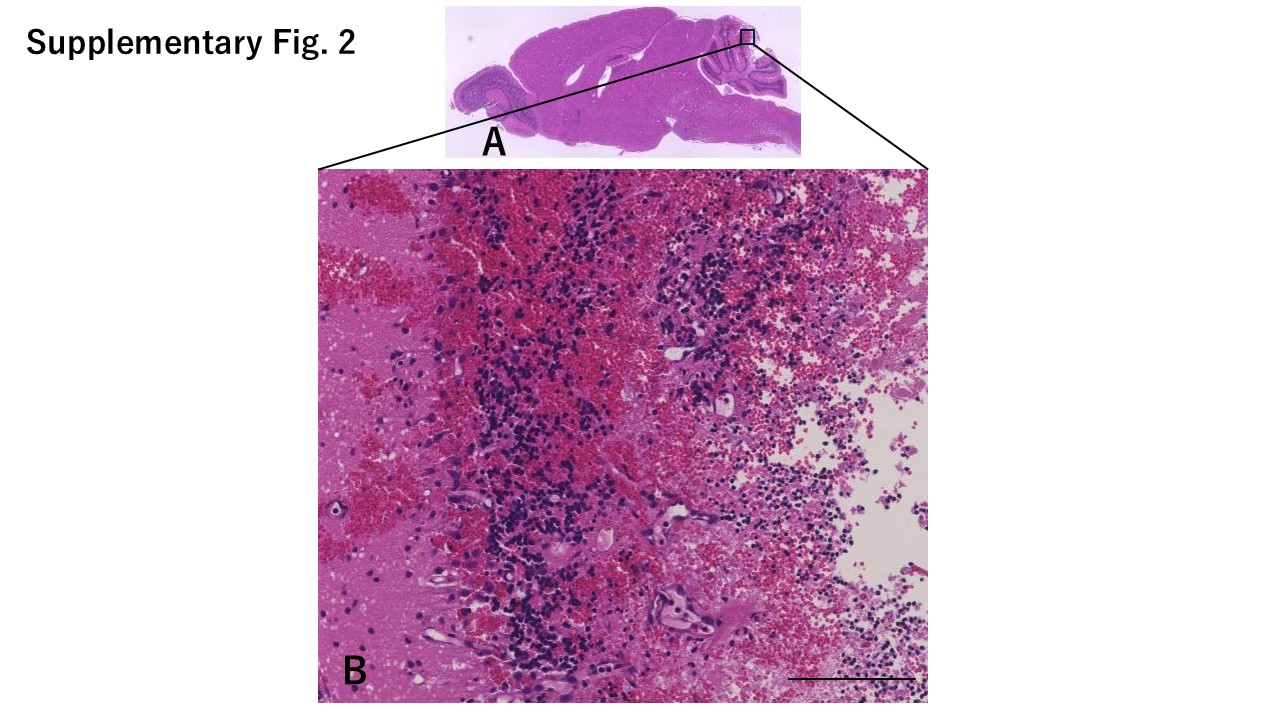

Supplement: Supplementary file 2 [file Image_2.jpeg]

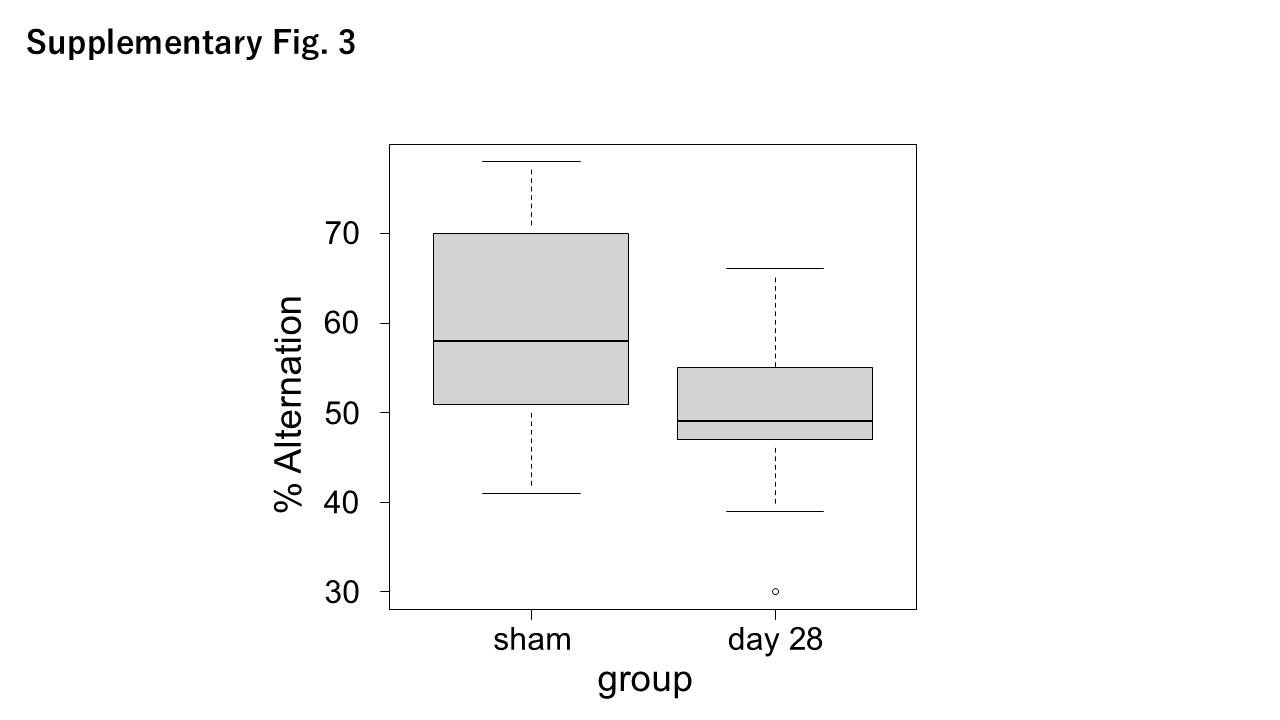

Supplement: Supplementary file 3 [file Image_3.jpeg]

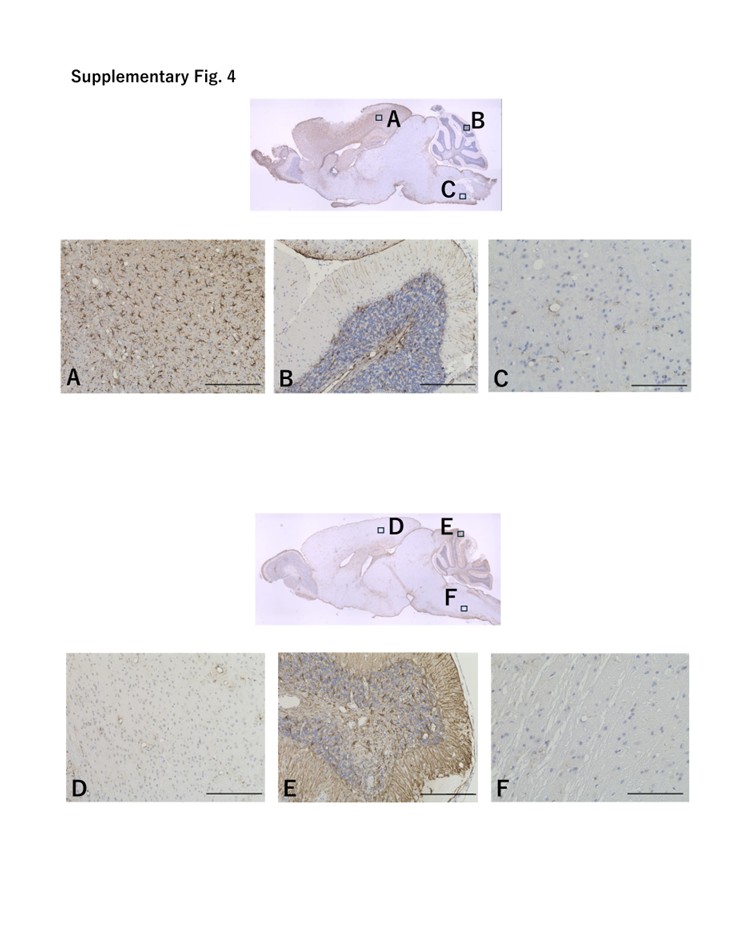

Supplement: Supplementary file 4 [file Image_4.jpeg]

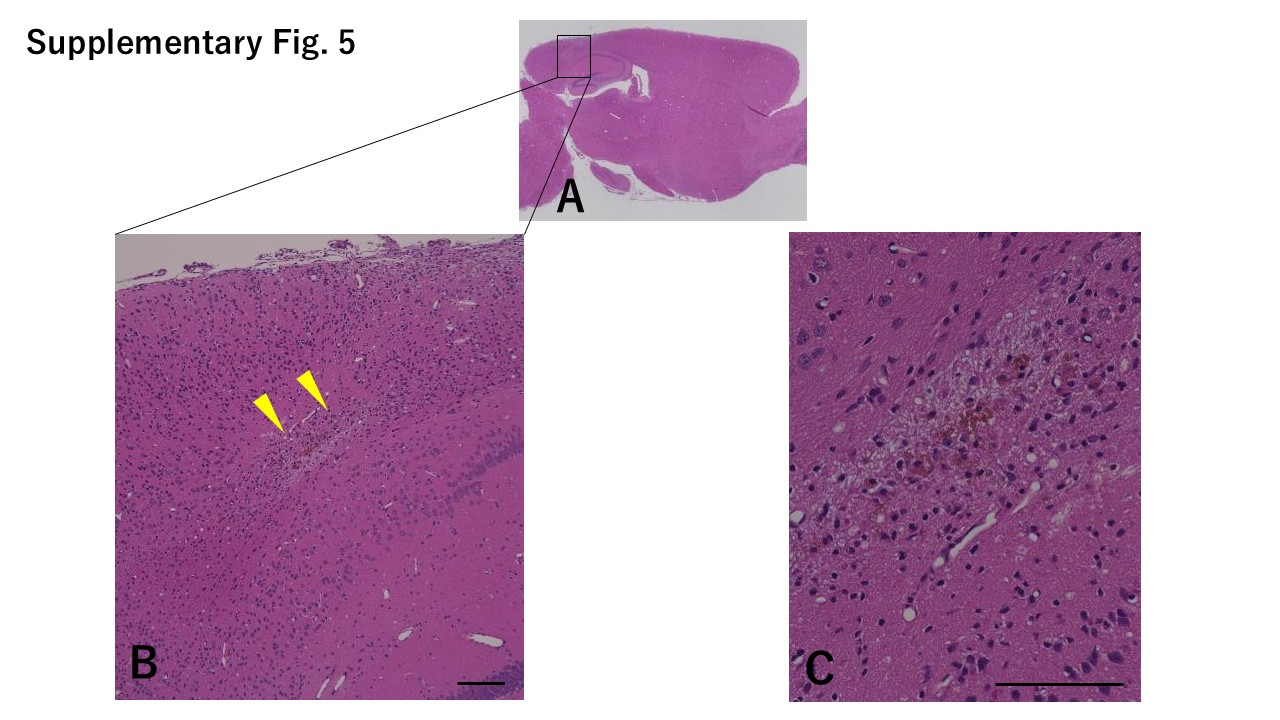

Supplement: Supplementary file 5 [file Image_5.jpeg]
