# Supplementary material for: Brainstem dysfunction induced by laser-induced shock wave results in hippocampal CA3 neuronal injury in mice
Source: Front Neurol. 2026 Jan 12;16:1722482. doi: 10.3389/fneur.2025.1722482 (PMC12832503; doi:10.3389/fneur.2025.1722482)
Supplement: Supplementary file 6 [file Supplementary_file_1.docx]

Supplementary Material

# Supplementary Data

**Supplementary Fig. 1**: Representative hematoxylin and eosin-stained coronal and sagittal sections of the hippocampus.

(**A**) Sagittal section taken 1 mm lateral to the midline; (**B**) Coronal section located 2 mm posterior to bregma. Comparable hippocampal morphology is observed in both sections.

**Supplementary Fig. 2:** Histopathological findings 3 days after LISW exposure to the upper neck region, shown in a sagittal brain section (hematoxylin and eosin staining). Scale bars = 100 µm. LISW, laser-induced shock wave.

(**A**) Hemorrhage and tissue contusion are visible in the dorsal cerebellum.

(**B**) High-magnification view of the contused region indicated by the box in panel A, showing petechial to patchy hemorrhages surrounding the contusion core. Scale bar = 100 µm.

**Supplementary Fig. 3:** Quantification of the % Alternation in the sham and day 28 groups.

The day 28 group showed a lower trend in % Alternation compared with the sham group (50 ± 10% vs. 59 ± 12%, respectively; *p* = 0.08), suggesting a potential decline in short-term spatial working memory.

**Supplementary Fig. 4:** Representative GFAP immunoreactivity in median sagittal brain sections following LISW exposure to either the parietal or upper neck region on day 3. The LISW exposure to the parietal region was performed on the left side, 2 mm lateral and 2 mm rostral to the midline connecting both ears. The fluence, impulse, and focal target were identical to those used for LISW exposure to the upper neck region. Scale bars = 100 µm. GFAP, glial fibrillary acidic protein; LISW, laser-induced shock wave.

**(A)** GFAP immunoreactivity in the parietal lobe after LISW exposure to the parietal region. Marked increase in astrocytic activation was observed in the parietal cortex, indicating astrocytic activation at the direct impact site.

**(B)** GFAP immunoreactivity in the dorsal cerebellum after LISW exposure to the parietal region. No increase in astrocytic activation was observed, indicating that the shockwave did not propagate to the posterior brain structures.

**(C)** GFAP immunoreactivity in the brainstem after LISW exposure to the parietal region. No increase in astrocytic activation was observed.

**(D)** GFAP immunoreactivity in the parietal lobe after LISW exposure to the upper neck region. No increase in astrocytic activation was observed, indicating no direct injury to the parietal cortex.

**(E)** GFAP immunoreactivity in the dorsal cerebellum after LISW exposure to the upper neck region. Astrocytic activation was significantly increased, indicating that shockwave energy propagated toward the posterior structures, including the cerebellum.

**(F)** GFAP immunoreactivity in the brainstem after LISW exposure to the upper neck region. No increase in astrocytic activation was observed.

**Supplementary Fig. 5:** Representative hematoxylin and eosin-staining showing hemosiderin deposition near the left CA3 region. Scale bars = 100 µm.

**(A)** Sagittal section stained with hematoxylin and eosin, obtained 1 mm left lateral to the midline.

**(B)** Higher-magnification image (×10) of the parietal region, including the left CA3 area. Arrows indicate hemosiderin deposition adjacent to the CA3 region.

**(C)** High-magnification image (×20) highlighting hemosiderin deposition.
